# Adaptation and validity of the Trait Meta-Mood scale for Brazilian adolescents

**DOI:** 10.3389/fpsyg.2023.1058426

**Published:** 2023-02-06

**Authors:** Sheila Gonçalves Câmara, Mary Sandra Carlotto, Rosario Cabello, Pablo Fernández-Berrocal

**Affiliations:** ^1^Graduate Program of Psychology and Health, Department of Psychology, Federal University of Health Sciences of Porto Alegre, Porto Alegre, Brazil; ^2^National Association for Research and Postgraduate Studies in Psychology (ANPEPP), Porto Alegre, Brazil; ^3^Department of Developmental and Educational Psychology, Faculty of Psychology, University of Málaga, Málaga, Spain; ^4^Department of Basic Psychology, Faculty of Psychology, University of Málaga, Málaga, Spain

**Keywords:** adolescents, test validity, factor analysis, emotional intelligence, life satisfaction, mental health

## Abstract

The current study aimed to present the process of adaption and validity evidence of the *Trait Meta-Mood Scale* (TMMS-24) for the population of Brazilian school adolescents. The study included 4,681 students aged 10 to 19 years old, attending public schools in 12 cities in the state of Rio Grande do Sul, Brazil. According to the original scale, the exploratory and confirmatory factorial analyses indicated adequate fit indexes and satisfactory reliability for the model composed of 24 items and three factors (attention, clarity, and repair). The scale showed a significant positive correlation with life satisfaction and psychological well-being. The results reveal that the TMMS-24 is adequate for the assessment of emotional intelligence among Brazilian adolescents and may contribute to research and interventions regarding emotional competencies among this population.

## 1. Introduction

Emotional intelligence (EI) is a set of skills that explains the use of emotions for more effective reasoning by providing a unified understanding of cognitive and emotional abilities (Mayer and Salovey, 1995). Research on EI has shown that it consists of a protective factor for health that impacts well-being and stress (Puigbó et al., 2019). Specifically, EI is defined as the integration of several capacities: “the ability to perceive, evaluate, and express emotions accurately; the ability to access and/or generate feelings when they facilitate thinking; the ability to understand emotion and emotional knowledge; and the ability to regulate emotions to promote emotional and intellectual growth” (Mayer and Salovey, 1997, p. 10).

Studies on EI have increased significantly over the years, and several theories, models, and instruments for its assessment have been developed (Joseph and Newman, 2010; Fernández-Berrocal et al., 2019). Joseph and Newman (2010) proposed three models, which can be distinguished according to the type of measurement instruments that were employed: (1) the mixed self-report model considers EI a broad concept that includes (among others) motivations, interpersonal and intrapersonal skills, empathy, personality factors, and well-being (Mayer et al., 2008), and uses self-report instruments to assess participants’ subjective perception; (2) the performance-based skills model, in which EI is viewed as a form of intelligence that involves reasoning about our emotions (Mayer et al., 2016) and is assessed by emotional problem solving, using performance tests that include a set of correct and incorrect answers; and (3) the self-report skill model, which, like the performance-based skill model, views EI as a combination of emotional abilities; in this case, however, self-report instruments are used, in which participants must estimate their own EI subjectively (Extremera and Fernández-Berrocal, 2005).

The trait meta-mood scale (TMMS; Salovey et al., 1995) is an ability self-report model composed of three dimensions: attention to feelings, clarity of feelings, and emotion repair, which are organized hierarchically. The attention process comes first, followed by the clarity process, and finally, the emotion repair process. Therefore, the base of the hierarchical structure concerns the ability to recognize feelings and their meanings. Clarity corresponds to the ability to know and understand one’s own emotions identified in the previous process. Then, from the recognition and understanding of emotions, the last process—repair—allows for the control of emotions, both positive and negative, and to use them to achieve the most appropriate emotional level and intensity for each situation.

Emotional intelligence has potential health benefits, both physical and mental. In terms of mental health, it is essential to consider the relationship between EI and other psychological variables, not only the negative ones but also those indicative of positive mental health. Among these, the literature has pointed to life satisfaction and psychological well-being (Salguero et al., 2012;Llamas-Diaz et al., 2022).

Emotional intelligence develops during human development (Cabello et al., 2016). According to a definition established by the World Health Organization (WHO), the chronological period of adolescence is between 10 and 19 years (Organização Mundial da Saúde, 2015). The stage is one of the shortest in human development and events related to it have a severe impact on adolescents’ health, identity construction, and development (Vega et al., 2021), with significant effects on their health in adulthood (Dick and Ferguson, 2015).

Studies on EI among adolescents show that an adequate level of EI can act as a protective factor for psychosocial adjustment (Inglés et al., 2015). In the same vein, other studies have shown that EI is associated with decreased aggressive behavior, such as physical and verbal aggression (Vega et al., 2021) or suicide risk (Domínguez-García and Fernández-Berrocal, 2018). In a more positive direction, a recent meta-analysis on the relationship between EI and well-being in adolescents demonstrates that the ability to perceive, use, understand and regulate emotions in oneself and others relates to adolescents’ subjective well-being at both cognitive and affective levels (Llamas-Diaz et al., 2022).

In this sense, the availability of instruments on EI in adolescents allows us to contemplate developmental characteristics in the psychological, emotional, educational, and social spheres. Specifically, instruments designed for this population allow for a better understanding and adequacy according to their reality and provide an approximation of adolescents with their affections and a sense of their emotions.

### 1.1. Evaluation of the *Trait Meta-Mood Scale*

Different reviews have shown that among the existing instruments to assess EI in adolescents, the Trait Meta-Mood Scale (TMMS), in different versions, has been one of the most widely used to evaluate EI skills in adolescents (Batista and Noronha, 2018; Llamas-Diaz et al., 2022).

The original version consists of 48 items covering three factors: attention, clarity, and repair (Salovey et al., 1995). The same group that developed the TMMS, after evaluating and removing items that presented low internal consistency, proposed a version of 30 items distributed in the same factorial structure as the larger version (Salovey et al., 1995), presenting adequate indices of internal consistency in versions for Italia (Giromini et al., 2017), Korea (Lee and Lee, 1997), Germany (Otto et al., 2001), and China (Li et al., 2002).

In Spain, Fernández-Berrocal et al. (2004) conducted a new study with the TMMS to update the scale and refine the psychometric measures. The authors developed a reduced version of the scale, keeping the same three-factor structure of the original scale, which was named TMMS-24. The new version was due to the low reliability of the instrument in the Spanish version. Items were eliminated due to their low contribution to the reliability of the instrument and by semantic analysis of their content so that items assessing emotional aspects in general or that indicated the assessment of interpersonal emotional skills rather than intrapersonal EI were excluded. Another change was in the wording of the negative items, which were worded positively to improve their comprehension in the Spanish population (Fernández-Berrocal et al., 2004; Queirós et al., 2005).

Principal components analysis and Varimax rotation were performed with data from a previous study in which the TMMS-48 had been used (Fernandez-Berrocal et al., 1998). Items with a factor loading of 0.40 or less were eliminated, setting up the version of 24 items and three factors, each with eight items. Internal consistency was satisfactory in all three factors; test–retest correlations after the four-week period were high; the three factors also correlated in the expected direction with the instruments assessing depression, rumination, and life satisfaction (Fernández-Berrocal et al., 2004).

In 2005, the TMMS-24 was validated for the Portuguese population by Queirós et al. (2005). The scale showed stability and good reliability in the three-factor structure, and significant relationships in the expected direction with the criterion variables considered: depression scale, ruminative response scale, life satisfaction scale, and mental health. In Mexico, a study on the TMMS-24 with 6,105 university students analyzed a one-factor model of EI and a three-factor model, which showed better fit and internal consistency indices (Valdivia Vázquez et al., 2014). A recent study comparing Spanish-speaking countries (Spain, Argentina, and Ecuador) with 1,048 adults found adequate psychometric properties for all countries and demonstrated the three-factor invariance of TMMS-24 (Górriz et al., 2021). Another study with Argentinian university students (*n* = 374) found satisfactory psychometric properties, demonstrating that the most appropriate factor structure is the three-factor one (González et al., 2021).

### 1.2. Evaluation of the Trait Meta-Mood scale-24 in adolescents

Regarding the adolescent population, Salguero et al. (2010) assessed 1,497 Spanish schoolchildren using the TMMS-24, corroborating the original model of the scale. One item was eliminated in the repair dimension, leaving the scale with 23 items. In 2014, a study with 2,693 Spanish adolescents found similar results, but the authors kept the 24-item structure (Pedrosa et al., 2014). A study including 1,038 adolescents was conducted using a short version of the TMMS-23 in the Basque Country. Confirmatory factor analysis was consistent with the original TMMS model (Salovey et al., 1995), internal consistency indices were adequate, and temporal stability was verified in all dimensions (Gorostiaga et al., 2011).

An Argentine study developed its own version of the TMMS based on theory and previous instruments. Data were collected from 400 adolescents. The results were consistent with the original distribution of the TMMS, and the version composed of 21 items with satisfactory reliability coefficients (Calero, 2013). An adaptation of the TMMS-24 was conducted in Chile with a sample of 3.255 adolescents. The three-factor structure with 24 items presented a model with good fit indices (Gómez-Núñez et al., 2018).

### 1.3. Present study

The studies on the TMMS, especially the TMMS-24, with the general population and with adolescents, consider that it is a valid and reliable instrument to be assessed in the Brazilian context. Given the scarcity of EI assessment instruments among adolescents in Brazil, this study aimed to evaluate the validity of evidence of the TMMS-24 among Brazilian adolescents.

Specifically, considering the previous scientific evidence, we propose the following hypothesis (1) we anticipate finding the theoretical proposal (Salguero et al., 2010; Brito-Costa et al., 2016) of a three-dimensional factor structure model of the Brazilian version of the TMMS-24 *via* Confirmatory Factor Analysis (CFA); (2) we expect, like as previous studies (Queirós et al., 2005; Sanchis-Sanchis et al., 2020), that this factor structure of the TMMS-24 will be invariance across sexes; (3) we await good reliability of the Brazilian version of the TMMS-24 to three dimensions; (4) finally, considering previous studies that evaluated the relationship between the dimensions of the TMMS-24 and psychological well-being and life satisfaction (Hodzic et al., 2016; Guasp Coll et al., 2022; Llamas-Diaz et al., 2022), we expect to show a convergent validity of the Brazilian version of the TMMS-24 through correlations between the TMMS-24 scores with mental health and life satisfaction.

## 2. Materials and methods

The present cross-sectional study followed the guidelines of International Test Commission (2016) in terms of the stages of adaptation and analysis of the validity evidence of the TMMS-24. When the interest in adapting the TMMS-24 for Brazilian adolescents emerged, contact was made with the researcher and first author of the article “Validity and Reliability of the Spanish modified version of the Trait Meta-Mood Scale” by Pablo Fernández-Berrocal from the University of Malaga, Spain. The author responded on behalf of himself and other colleagues in the study, expressing his agreement to adapt the instrument in Brazil and forwarding support materials for the Brazilian study.

### 2.1. Translation and adaptation of the Trait Meta-Mood Scale

We used the Spanish, European Portuguese, and English versions of the TMMS-24 (only the items equivalent to the reduced version of 24 items). The Spanish and English versions were translated into Portuguese by two literature professors, each trained in the language of the version to be translated. The Portuguese versions were back-translated into their original languages by two bilingual social psychologists, in Brazilian Portuguese and in the language to be back-translated.

The adaptation and evaluation of the items of the Portuguese version was conducted by the researchers, considering: (1) conceptual and item equivalence; the equivalences were evaluated by four experts in social psychology; the items were evaluated in terms of their connotation in relation to the original terms, conceptual adequacy in Portuguese and Brazilian cultures, representativeness of the items regarding the domain corresponding to the instrument, and conceptual adequacy regarding the age range of the target population; (2) semantic equivalence; the items were assessed through an adaptation from Portuguese of Portugal to Brazilian Portuguese by two psychologists knowledgeable in both cultures; the items were modified according to the comparison of the two versions obtained; and (3) operational equivalence, obtained by maintaining the same number of questions and response options used in the original instrument; self-completion format and standardization regarding data collection (Reichenheim and Moraes, 2007; International Test Commission, 2016).

To assess the quality of the translation concerning language clarity, practical relevance, and theoretical relevance, the Content Validity Index (CVC) was calculated. The CVC of language clarity was 0.93, practical relevance 0.96, and theoretical relevance 0.96. The overall CVC was 0.98. No item had a CVC index lower than 0.80, indicating good content validity results in the instrument’s translation stage (Hernandéz-Nieto, 2002). For the theoretical dimension, as it is a categorical variable, the kappa coefficient was used, which was 0.86, a value that can be considered solid (Landis and Koch, 1977).

### 2.2. Evidence of content validity of traiTrait Meta-Mood Scale

The three versions obtained, together with the versions in the source language, were evaluated by two social psychologists, who independently compared the versions and evaluated: (1) the items in terms of their connotation in relation to the original terms, (2) the conceptual adequacy of the terms in the cultures covered, (3) the representativeness of the items as to the domain corresponding to the instrument, and (4) the conceptual adequacy as to the age group of the target population (adolescents).

After adjustments were made to the three Brazilian Portuguese versions, they were compared and integrated, giving rise to a unified version of the instrument. Three other social psychologists analyzed the resulting version, contributing suggestions about the terms used and the classification of the items related to the proposed factors. Four items were modified since more than 80% of the judges agreed that they were not clear to the study population (items 4, 6, 22, and 24, which used the term “disposition”). As for the classification of the items in their original factors, there were no discrepancies in more than 80% of the evaluations.

The instrument obtained was submitted to semantic analysis by six adolescent students from a public school in Porto Alegre, Brazil. Of these, three were girls and three were boys, aged between 12 and 15 years. A group meeting was held in which a researcher read the instrument together with the adolescents. After reading each item, the participants expressed their understanding, explaining what they had understood of each statement. There were no difficulties in understanding and responding on the part of the adolescents.

### 2.3. Trait Meta-Mood Scale pilot study

The instrument was applied to 163 school adolescents, students from fifth to ninth grades of elementary school, from the public network of a city in the metropolitan region of Porto Alegre, Brazil. The collection was conducted in two schools, one in a more vulnerable region of the city and the other in the central region. Of these, 87 (53.4%) were girls. The ages ranged from 10 to 18 years.

The distribution of responses for each item across the 5 points of the response scale was assessed. No “floor” or “ceiling” effects were identified. Means ranged from 2.56 (item 14) to 3.69 (item 4); standard deviations ranged from 1.18 (items 18 and 24) to 1.32 (item 5); asymmetry values were less than ± 1.00 (Hair et al., 2014). The values of McDonald’s omega estimator were 0.90 for the attention and clarity factors, and 0.92 for the repair factor. Cronbach’s alpha coefficients were 0.93 for the attention factor, 0.89 for the clarity factor, and 0.90 for the repair factor, with all items contributing to reliability.

### 2.4. Evidence for construct validity, criterion validity, and reliability of the Trait Meta-Mood Scale

#### 2.4.1. Context of the study

The state of Rio Grande do Sul (RS) consists of 497 municipalities, divided into seven regions. The Metropolitan Region of Porto Alegre (MRPA), with 34 municipalities, is the largest metropolitan region in the South Region of Brazil, with approximately 4.4 million inhabitants, and the fifth most populous in the country. In RS it is the most densely populated area, concentrating 38.2% of the state’s total population. The population of school adolescents enrolled in public schools in Rio Grande do Sul, between 2013 and 2018, ranged from 336,435 to 284,890 (*M* = 312,850; SD = 23,167). In MRPA, in 2018, there were about 38,974 students enrolled in high school, which corresponds to the age group of the study (Rio Grande do Sul, Secretaria de Planejamento, Governança e Gestão, Departamento de Planejamento Governamental, 2021).

#### 2.4.2. Data collection procedures and participants

The data collection procedures were through two research projects. First, with the project “Contexts of life and health of school adolescents in southern Brazil” we contacted 77 schools in 11 municipalities of the state for approval. Second, the project “Prevalence and functions of non-suicidal self-mutilation among school-aged adolescents in the municipal public network of Montenegro/RS” was forwarded to the Municipal Secretariat of Education and Culture (SMEC) of the municipality, where the research was conducted, for appreciation and agreement to conduct the investigation. In this case, 10 schools were contacted, of which nine agreed to participate. Together, between both projects, the participation of 86 schools was achieved.

After the approval of the schools, both projects were submitted and approved by the research ethics committees. Data collection was conducted collectively, in classrooms, by one of the researchers and research volunteers duly trained for the activity. The average administration time was 30 min. The participants answered the research instrument with their express consent (Consent Form) and the authorization of those responsible who signed the Informed Consent Form.

From this data collection, we used information from 4,753 school adolescents, and elementary school students from 86 public schools in the Rio Grande do Sul, Brazil, who filled out the TMMS-24 between the years 2013 and 2018 in the projects. Specifically, data were collected in 12 cities in the state, mostly in Porto Alegre (*n* = 1,326; 28,3%). Sixty-three participants were excluded for not having answered at least 90% of the instrument, and nine were identified as extreme cases (outliers), detected by Mahalanobis’ D2. Thus, the final sample was composed of 4,681 participants. Of these, 2,475 (52.9%) were girls. Age ranged from 10 to 19 years (*M* = 14.2, SD = 1.35). As for the year of study, most participants were in ninth grade (*n* = 3,833; 81.88%); 245 (5.23%) were in eighth grade; 210 (4.49%) in seventh grade; 195 (4.17%) in sixth grade; and 198 (4.23%), in fifth grade.

All adolescents included in our study were collected in 12 cities in the Metropolitan Region of Porto Alegre. In terms of the population of adolescent students in RS, the study sample represents 1.5%. As for the population of students in the MRPA, it represents 12%. Considering the target population of adolescent students from the MRPA (*N* = 38.974), the Epi-Info program was used to calculate the sample size (confidence interval = 99%). The adopted parameters were a prevalence of 50%, and a design effect of 1.5. Thus, the calculated sample would be 993 adolescent students. Concerning MRPA, the sample is quantitatively and qualitatively representative. The data, however, cannot be extrapolated to the population of adolescent students from RS or Brazil, given their geographic configuration.

### 2.5. Instruments

1. Sociodemographic data survey: sex, age, and current grade.

2. *Trait meta-mood scale* (TMMS-24; Fernández-Berrocal et al., 2004). The scale consists of 24 items, divided into three factors: attention, clarity, and repair, with eight items per factor. These are evaluated according to the degree of agreement of the participants with each item using a five-point Likert-type scale, ranging from “strongly disagree” (1) to “strongly agree” (5). The reliability of the instrument, for each dimension, in the version developed by the authors, was: attention (α = 0.90); clarity (α = 0.90), and repair (α = 0.86; Fernández-Berrocal et al., 2004).

3. *Brief multidimensional students’ life satisfaction scale* (BMSLSS; Seligson et al., 2003). BMSLSS is a six-item measure whose sum of subjects’ scores provides an overall life satisfaction score. The items concern satisfaction with family, with friends, with the school experience, with oneself, with where one lives, and with life in general. The last item is considered a single item because it deals with overall satisfaction with one’s life. The answers range from very poor (0) to great (10). In Brazil, in a study on the validity evidence of the instrument, the scale achieved an internal consistency index of 0.72 (Bedin and Sarriera, 2014). In the present study, the scale presented α = 0.79.

4. *General health questionnaire* (*GHQ*-12; Goldberg and Williams, 1988). It consists of 12 items with responses on a 4-point scale. It assesses mental health over the previous 6 months using the mean of the answers to the 12 items, with lower means indicating better mental health. In a scale validation study assessing the psychological well-being of adolescents in southern Brazil, the GHQ-12 showed an internal consistency coefficient of 0.80 (Sarriera et al., 1996). In the present study, the alpha was 0.86.

### 2.6. Data analysis procedures

After unifying the databases, a univariate analysis was performed to describe the participants and the behavior of the items and dimensions of the scale in the study population. Evidence of construct validity was assessed by exploratory and confirmatory factor analysis. Exploratory factor analysis was performed using IBM SPSS v.25, by the principal axis extraction method, Oblimin rotation, to verify the factorial distribution of the instrument. The results were analyzed in terms of factor distribution and factor loadings. Parallel analysis was conducted with Factor, version 10.10.02, with a random permutation of sample values as the criterion for factor retention (Timmerman and Lorenzo-Seva, 2011).

Confirmatory factor analysis was performed using the Robust Weighted Least Squares estimation method, using Mplus®, version 6.2. This method was chosen because it is the most appropriate in the case of non-normal distribution responses, besides the fact that the scale responses were ordinal, Likert scale responses (Wang and Wang, 2019).

As parameters for a satisfactory model fit, we considered the comparative fit index (Bentler’s Comparative Fit Index, CFI) with values greater than 0.95 (Wang and Wang, 2019) and residuals by Root Mean Square Error of Approximation (RMSEA) with an expected value below 0.08, considering a 90% confidence interval; Byrne, 2016). First-order models were tested, with a correlation between the three latent factors of the TMMS-24, and second-order models, considering the emotion regulation construct (Gross and Thompson, 2015).

A Multigroup Confirmatory Factor Analysis (MCFA) was also performed to verify the invariance of the TMMS-24 across sexes. The models of configural invariance (structure equivalence), metric invariance (factor loadings equivalence), and scalar invariance (equivalence of intercepts) were tested. Instrument invariance was assessed using the Comparative Fit Index (ΔCFI) difference test and the Standardized Root Mean Square Residual (SRMR), with an expected value less than 0.08, considering a 90% confidence interval; Byrne, 2016). Comparative fit indices between the models were evaluated, considering the difference between the CFI from one model to another (ΔCFI), which should not be greater than 0.01 (Milfont and Fischer, 2010). Reliability was assessed using Cronbach’s alpha (α) and McDonald’s omega estimator (ωt), which were used to overcome the limitations of α (Dunn et al., 2013).

Evidence of convergent validity was carried out considering previous studies that evaluated the relationship between the dimensions of the TMMS-24 and psychological well-being and life satisfaction (Hodzic et al., 2016; Guasp Coll et al., 2022), as well as the psychological well-being construct’ operationalization in the dimensions of life satisfaction and mental health (Hodzic et al., 2016). Pearson’s Correlation analyses were conducted to assess the relationship between the TMMS-24 dimensions with the BMSLSS and the GHQ-12 instruments. The BMSLSS aims to identify a multidimensional profile of children’s and adolescents’ judgments about their life satisfaction in terms of specific important domains in their lives (family, friends, student experience, self, and where they live), in addition to assessing their general satisfaction with life (Seligson et al., 2003). The GHQ-12 assesses mental health based on the functioning of individuals, comparing their current and usual functioning patterns. The elements are evaluated based on the interviewee’s recent experiences, focusing not on the problem but the changes recently observed by the individual in their behavior (Goldberg and Williams, 1988). A significance level of 95% (*p* ≤ 0.05) was adopted in all statistical procedures.

### 2.7. Ethical aspects

Both projects were conducted within the framework of the Declaration of Helsinki and submitted and approved by the Research Ethics Committee of the Universidade Luterana do Brasil (Contexts of life and health of school adolescents in southern Brazil, under approval no. 132.167) and the Research Ethics Committee of the Universidade Federal de Ciências da Saúde de Porto Alegre (Prevalence and functions of non-suicidal self-mutilation among school adolescents from the municipal public network of Montenegro/RS, approval number 2.971.591). The participating adolescents were informed of the study’s objectives, signed the consent form, and brought the Informed Consent Form signed by their parents/guardians. The ethical precepts for research with human beings were respected during the research, as recommended by the Brazilian National Health Council, Resolution Number 466/12 (Brasil, 2012).

## 3. Results

### 3.1. Exploratory factor analysis

Exploratory factor analysis showed a three-factor distribution according to the eigenvalues of the factors. Parallel analysis, with a random permutation of sample values, corroborated the retention of three factors (Timmerman and Lorenzo-Seva, 2011). Sample adequacy showed the following indices: Kaiser-Meyer-Olkin (KMO = 0.946) and Bartlett’s test of sphericity (x^2^ = 35,881; *p* ≤ 0.01). The determinant of the correlation matrix (0.00046) was greater than 0.00001, indicating no multi-collinearity among the items (Field, 2020). Exploratory factor analysis showed an explained variance of 45.9%, with three factors and the same distribution of items per factor as the original scale. Factor loadings ranged from 0.356 to 0.667 (attention factor), 0.434 to 0.666 (clarity factor), and 0.423 to 0.681 (repair factor; see Table 1).

**Table 1:** Structure matrix of the TMMS-24 in the exploratory factor analysis.

TMMS -24 Items	Factors		
	Attention	Repair	Clarity
IE1	**0.651**	0.367	0.481
IE2	**0.684**	0.337	0.443
IE3	**0.601**	0.322	0.408
IE4	**0.601**	0.345	0.390
IE5	**0.374**	0.075	0.197
IE6	**0.538**	0.295	0.317
IE7	**0.705**	0.339	0.450
IE8	**0.646**	0.428	0.596
IE9	0.446	0.434	**0.665**
IE10	0.424	0.439	**0.696**
IE11	0.406	0.394	**0.652**
IE12	0.411	0.416	**0.591**
IE13	0.415	0.473	**0.579**
IE14	0.373	0.416	**0.495**
IE15	0.339	0.377	**0.466**
IE16	0.441	0.527	**0.585**
IE17	0.358	**0.604**	0.491
IE18	0.321	**0.722**	0.468
IE19	0.284	**0.706**	0.437
IE20	0.337	**0.721**	0.463
IE21	0.329	**0.628**	0.451
IE22	0.414	**0.608**	0.451
IE23	0.426	**0.583**	0.524
IE24	0.281	**0.458**	0.375

Items loading in dimensional factor are bolded.

### 3.2. Confirmatory factor analysis

**Figure 1:**
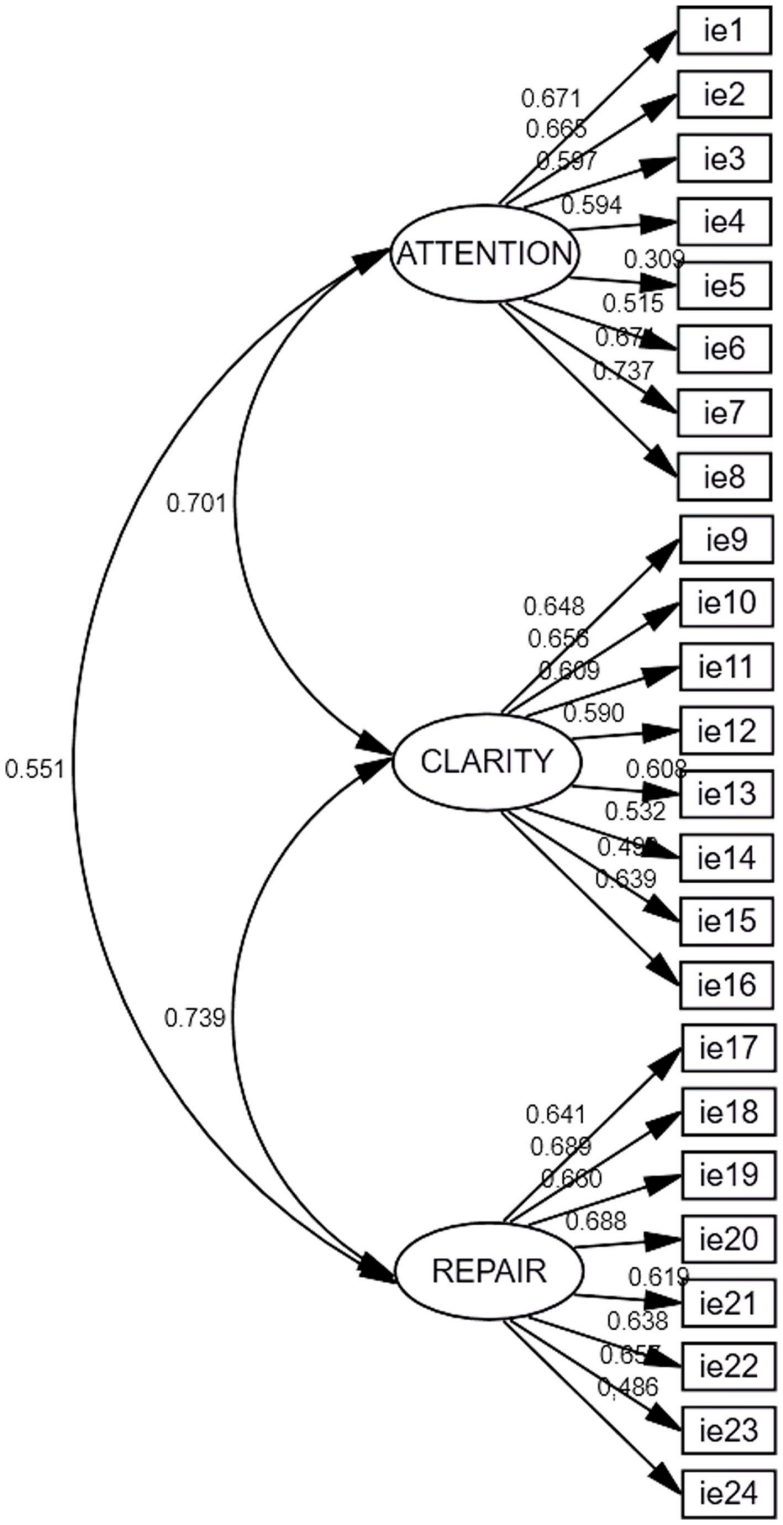
CFA of the final 1st order three-factor TMMS-24 model.

The confirmatory factor analysis evaluated the theoretical proposal of a three-dimensional factor structure model. All factor weights (Lambdas –λ) were positive and statistically different from zero, with values between 0.309 and 0.737. The raw scores of the three factors showed correlations with each other, ranging between 0.551 and 0.701. The model showed satisfactory indexes, with CFI values close to those recommended. Items 5, 15, and 24 showed factor loadings lower than 0.500. The analysis of the modification indices indicated the possibility of correlation between the errors of items 9 and 10, 5 and 6, and 18 and 19 for a better model fit. However, it was decided not to exclude items with low factor loadings or perform *post-hoc* modifications and keep the original. The model showed: χ2 = 3,218,446 (*p* < 0.01); df = 249; CFI = 0.931; RMSEA = 0.050 (CI = 0.049–0.052) and SRMS = 0.036. The model is shown in Figure 1.

In line with studies on sex differences in emotion regulation (Silk et al., 2003; Nolen-Hoeksema and Aldao, 2011) and the distinct research parameters of each dimension of the TMMS-24 according to sex (Queirós et al., 2005), an MCFA was performed to assess the configural, metric, and scalar invariance of the instrument. The models showed no worsening in CFI indices greater than 0.01 compared to the previous model. The results regarding RMSEA and SRMR were satisfactory (Cheung and Rensvold, 2002). The results showed evidence that the structure is stable and that there are no response biases to TMMS-24 related to sex (Table 2).

**Table 2:** Indicators produced by the multi-group confirmatory factor analysis for factorial invariance tests between sex (RS, 2013–2018; *n* = 4.681).

Model	χ^2^	Gl	*p*	CFI	RMSEA (IC)	SRMR
Model 1: Configural	3,590,32	498	<0.01	0.93	0,02 (0,02−0,03)	0,0399
Model 2: Metric	3,595,52	519	<0.01	0.93	0,02 (0,02−0,03)	0,0395
Model 3: Scalar	3,598,53	525	<0.01	0.93	0,02 (0,02−0,03)	0,0396

### 3.3. Descriptive statistics

The means of the items ranged from 1.29 to 4.13, with standard deviations between 0.76 and 1.29. Therefore, it can be stated that there was no floor or ceiling effect on any of the items. The repair dimension obtained the highest mean (*M* = 3.68; SD = 0.78), followed by the attention dimension (*M* = 3.56; SD = 0.76). The clarity dimension presented the lowest mean (*M* = 3.53; SD = 0.74). The asymmetry values were all less than ±1.00 (Hair et al., 2014); only item 23 showed an asymmetry value of −1.14. As for the item-total correlations, the values ranged from *r* = 0.31 to *r* = 0.58 in the attention dimension, *r* = 0.40 to *r* = 0.55 in the clarity dimension, and *r* = 0.39 to *r* = 0.59 in the repair dimension (Table 3).

**Table 3:** Descriptive statistics and internal consistency of the items and dimensions of the TMMS-24 (RS, 2013–2018; *n* = 4.681).

Dimension/Item	M (SD)	Corrected item-total correlation	Assime-tria	Alfa if item deleted	Ômega if item deleted
**Attention**					
1. Attention to feelings	3.70 (1.18)	0.52	−0.063	0.75	0.76
2. Preoccupation with feelings	3.73 (1.16)	0.56	−0.63	0.75	0.75
3. Usefulness of thinking about emotions	3.64 (1.20)	0.49	−0.58	0.76	0.77
4. To pay attention to emotions	3.68 (1.17)	0.49	−0.61	0.76	0.76
5. Interference feelings thoughts	3.05 (1.29)	0.31	−0.08	0.79	0.79
6. To think state of mind	3.24 (1.25)	0.44	−0.22	0.76	0.77
7. To think feelings	3.68 (1.19)	0.58	−0.59	0.74	0.75
8. Attention to feelings	3.75 (1.15)	0.52	−0.69	0.75	0.76
Total	3.56 (0.76)		−0.67	α = 0.79	ωt = 0.79
**Clarity**					
9. Knowledge about feelings	3.67 (1.17)	0.52	−0.60	0.75	0.75
10. To know what you feel	3.55 (1.19)	0.55	−0.47	0.75	0.74
11. To know exactly what you feel	3.55 (1.19)	0.53	−0.48	0.75	0.75
12. To recognize feelings	3.62 (1.15)	0.49	−0.50	0.76	0.76
13. Awareness about feelings	3.55 (1.15)	0.48	−0.46	0.76	0.76
14. Always say what you feel	3.26 (1.22)	0.43	−0.22	0.77	0.77
15. Sometimes say what you feel	3.42 (1.18)	0.40	−0.33	0.77	0.77
16. To understand what you feel	3.66 (1.15)	0.48	−0.52	0.76	0.76
Total	3.53 (0.74)		−0.67	α = 0.79	ωt = 0.78
**Repair**					
17. Optimistic attitude	3.67 (1.19)	0.49	−0.59	0.79	0.79
18. To think about nice things	3.73 (1.19)	0.59	−0.64	0.77	0.77
19. Annoyance nice things	3.51 (1.26)	0.58	−0.43	0.77	0.77
20. Positive thoughts	3.73 (1.19)	0.59	−0.64	0.77	0.77
21. To calm down	3.60 (1.22)	0.53	−0.52	0.78	0.78
22. Good state of mind	3.65 (1.18)	0.51	−0.52	0.78	0.79
23. Energy	4.13 (1.04)	0.48	−1.14	0.79	0.79
24. Change of disposition	3.47 (1.25)	0.39	−0.39	0.80	0.80
Total	3.68 (0.78)		−0.67	α = 0.81	ωt = 0.81

Reliability, assessed by Cronbach’s alpha coefficient, was 0.79, 0.79, and 0.81 for the Attention, Clarity, and Repair dimensions, respectively. Evaluation by McDonald’s omega estimator identified reliability of 0.92 for the whole scale, 0.79 in the Attention dimension, 0.78 in the Clarity dimension, and 0.71 in the Repair dimension (Table 3).

To analyze the evidence of convergent validity, initially, the means of the scales under study were transformed into z scores. The descriptive analysis and reliability indices of all scales in the study were calculated (Table 4). Next, we compared the means of the TMMS-24 dimensions (attention, clarity, and repair) with the means of five items of specific domains of life satisfaction and the overall life satisfaction item of the BMSLSS, and with the overall mean of the GHQ-12 (Table 5). The results of the correlations were in the expected direction. All means of the scales used as criteria showed low to moderate correlation with the dimensions of TMMS-24 (all *p* ≤ 0.01).

**Table 4:** Descriptive analysis and reliability indices of the scales in the study.

	*M*	DP	Range	Skewness	Kurtosis	Alpha	Omega	CRC	AVE
TMMS–Attention	3.56	0.76	1–5	−0.67	0.72	0.79	0.79	0.82	0.37
TMMS–Clarity	3.54	0,74	1–5	−0.63	0.64	0.79	0.78	0.82	0.36
TMMS–Repair	3.68	0.78	1–5	−0.67	0.46	0.81	0.81	0.84	0.41
BMSLSS domains	7.87	1.45	0–10	−1.29	2.62	0.72	0.77	–	–
BMSLSS overall life	7.65	2.59	0–10	−1.24	0.60	Unique item		–	–
GHQ-12	1.75	0.54	1–4	1.27	1.50	0.86	0.86	–	–

**Table 5:** Correlations between the means of the three dimensions of TMMS-24 and the BMSLSS and the GHQ-12 (RS, 2013–2018; *n* = 4.681).

	1	2	3	4	5	6
1. Attention	1					
2. Clarity	0.542**	1				
3. Repair	0.425**	0.597**	1			
4. BMSLSS domains	0.111**	0.176**	0.172**	1		
5. BMSLSS overall life	0.179**	0.197**	0.185**	0.529**	1	
6. GHQ-12	−0.081**	−0.332**	−0.374**	−0.306**	−0.298**	1

**p ≤ 0.01. BMSLSS, Brief Multidimensional Students’ Life Satisfaction Scale; GHQ-12, general health questionnaire.

## 4. Discussion

### 4.1. Resource identification initiative

The present study aimed to adapt and study the validity of the TMMS-24 for Brazilian adolescents. The results corroborated the hypothesis of the three-factor model (Salovey et al., 1995; Fernández-Berrocal et al., 2004), presenting adequate results with evidence of validity and reliability for its use in the Brazilian context.

The instrument’s adaptation process did not present greater difficulty due to the methodology adopted, based on the ITC guidelines (2016). For translation and back-translation, it was possible to use different versions of the TMMS-24 (Spanish, English, and Portuguese). In addition, the scale has a simple and objective structure for formulating the items. The cultural equivalence analysis showed that the three dimensions of TMMS-24 are appropriate, and the attributes used in the original version of the scale (Spanish) are equally valid for the target population (Reichenheim and Moraes, 2007). In terms of conceptual equivalence, a few items required adjustments.

Respect to our first hypothesis, the items were freely distributed into three factors in the exploratory factor analysis, correlated to the original instrument, without multicollinearity (Field, 2020). This step, using parallel analysis, has demonstrated the behavior of the instrument in the target population and, in addition, corroborated the three-factor distribution (Salguero et al., 2010; Brito-Costa et al., 2016). In this sense, the confirmatory factor analysis indicated that both the scale model with 24 items and three independent factors presented adequate fit indices for the sample under study. With these values, it can be concluded that the structural equation model showed an overall fit to the observed data and corroborated the instrument’s theoretical model (Salovey et al., 1995; Fernández-Berrocal et al., 2004). While the CFI is noticeably lower than 0.95, this value is quite close to the recommended value (Wang and Wang, 2019). Although the factor loadings of items 5, 15, and 24 were less than 0.500 and the modification indices indicated a better model fit to the error correlation of some items, the choice was to keep all 24 items of the scale and not perform *post-hoc* modifications to maintain the original structure of the scale. In addition, the items in question contributed to the internal consistency in their original dimension. The final model with the three latent variables observed—attention, clarity, and repair—showed satisfactory fit indices, mostly with factor loadings above 0.500, and the three latent variables correlated positively and significantly. These results were similar to those found in the studies of Fernández-Berrocal et al. (2004), Queirós et al. (2005), Pedrosa et al. (2014), and Gómez-Núñez et al. (2018) in Spain, Portugal, and Chile, respectively.

As for the correlation between factors, clarity and repair showed the highest correlation, followed by attention and clarity, and attention and repair. These results allow us to consider the procedural characteristic of EI since clarity regarding feelings is the second factor, which is related to the previous (attention) and subsequent (repair) ones (Caruso et al., 2015; Gross and Thompson, 2015).

To explore our second hypothesis, an MCFA was conducted to assess the configural, metric, and scalar invariance of the instrument because the survey parameters of each dimension of the TMMS-24 are different between men and women (Queirós et al., 2005; Sanchis-Sanchis et al., 2020). The results presented evidence that the structure is stable and that there are no response biases to the TMMS-24 between the sexes. The psychometric parameters of the scale are equivalent between boys and girls.

In the sample under study, the descriptive statistics of the TMMS-24 showed fairly close means among the dimensions of attention, clarity, and repair, all three being widely used. Responses to each item showed normal distribution, which was corroborated by asymmetry values (Hair et al., 2014). The corrected item-total correlations indicated that each scale dimension could be considered with a linear function of its component items (Tabachnick and Fidell, 2019).

To respond to our third hypothesis, we assessed the reliability of TMMS-24 by Cronbach’s alpha and McDonald’s omega (Table 3), and the reliability coefficients were satisfactory for each dimension (Tabachnick and Fidell, 2019). This, coupled with the semantic coherence of the set of items, allows us to consider that all items contribute to the establishment of the EI construct and its dimensions.

As for the convergent validity evidence, our fourth hypothesis, there were significant correlations between the three TMMS-24 dimensions and the comparison instruments BMSLSS (domains and overall life evaluation) and GHQ-12, according to the literature (Páez et al., 2006; Carmeli et al., 2009). The highest correlations were between TMMS-24 clarity and repair dimensions and the total BMSLSS and the GHQ-12. These results were also found in other studies that evaluated the relationship between EI with health and well-being (Salovey and Mayer, 1990; Mayer and Salovey, 1997; Carmeli et al., 2009). Correlations of the TMMS-24 and its dimensions were also significant and in the expected direction with overall life satisfaction and life domain satisfaction, as found in a recent meta-analysis (Llamas-Diaz et al., 2022).

The study has some limitations, such as the sample being composed only of adolescent students from southern Brazil. Another limitation is the low reliability of item 5, corresponding to the Attention dimension. Future studies should be analyzed if it depends on reading and/or emotional comprehension at this developmental stage. A final limitation is the use of databases from two research projects, which represented a time difference in data collection. The option of using these two databases was that the data collected were fully contemplated since both collections provided the study of the evidence of the validity of the instrument.

Further studies on the validity evidence of the TMMS-24 among Brazilian adolescents in different country regions may corroborate its functioning among adolescents from other Brazilian contexts. In the same sense, specific studies on the validity evidence of the TMMS-24 among adolescents in different countries may be helpful to have a tool for assessing emotional intelligence abilities from a transcultural perspective. Likewise, studies with out-of-school adolescents would help assess the reliability of the TMMS-24 since school is a protective factor for human development, which may represent differences between adolescents who are out of school.

The highlights of the study are the sample size and its coverage in several municipalities, although all are from southern Brazil. We also emphasize the use of two instruments as parameters for the evaluation of the convergent validity of the TMMS-24 in the target population. The data obtained allowed different procedures for analyzing the instrument, including allowing the identification of the stability of its structure in relation to sex in the population of Brazilian adolescents.

Based on the results obtained in this study, the TMMS-24 met the requirements for its use among Brazilian adolescents. This adaptation allows for the assessment of the EI process among adolescents, filling the gap in instruments suitable for this population. Its use in research, clinical, and school settings represents an additional resource for understanding and intervening in adolescent health and well-being.

## Data availability statement

The raw data supporting the conclusions of this article will be made available by the authors, without undue reservation.

## Ethics statement

The studies involving human participants were reviewed and approved by Research Ethics Committee of the Universidade Luterana do Brasil (contexts of life and health of school adolescents in sout`hern Brazil, under approval number 132.167) and the Research Ethics Committee of the Universidade Federal de Ciências da Saúde de Porto Alegre (prevalence and functions of non-suicidal self-mutilation among school adolescents from the municipal public network of Montenegro/RS, approval number 2.971.591). Written informed consent to participate in this study was provided by the participants’ legal guardian/next of kin.

## Author contributions

SC and MC: R&D project, data collection, analysis, and initial writing. RC and PF-B: R&D project, design, analysis, and co-writing the final draft. All authors contributed to the article and approved the submitted version.

## Funding

This research was partially supported by projects CTS-578 Junta de Andalucía and UMA18- FEDERJA-114 to PF-B and RC.

## Conflict of interest

The authors declare that the research was conducted in the absence of any commercial or financial relationships that could be construed as a potential conflict of interest.

## Publisher’s note

All claims expressed in this article are solely those of the authors and do not necessarily represent those of their affiliated organizations, or those of the publisher, the editors and the reviewers. Any product that may be evaluated in this article, or claim that may be made by its manufacturer, is not guaranteed or endorsed by the publisher.
